# Gut Microbial Signatures in Pediatric Crohn’s Disease Vary According to Disease Activity Measures and Are Influenced by Proxies of Gastrointestinal Transit Time: An ImageKids Study

**DOI:** 10.1093/ibd/izae199

**Published:** 2024-10-17

**Authors:** Ben Nichols, Richard K Russell, Bryn Short, Rodanthi Papadopoulou, Gili Focht, Umer Z Ijaz, Thomas D Walters, Malgorzata Sladek, Richard Hansen, David R Mack, Eytan Wine, Anne M Griffiths, Dan Turner, Konstantinos Gerasimidis

**Affiliations:** Human Nutrition, School of Medicine, Dentistry & Nursing, University of Glasgow, New Lister Building, Glasgow Royal Infirmary, Glasgow G31 2ER, UK; Department of Paediatric Gastroenterology, Royal Hospital for Children and Young People, 50 Little France Crescent, Edinburgh EH16 4TJ, UK; Human Nutrition, School of Medicine, Dentistry & Nursing, University of Glasgow, New Lister Building, Glasgow Royal Infirmary, Glasgow G31 2ER, UK; Human Nutrition, School of Medicine, Dentistry & Nursing, University of Glasgow, New Lister Building, Glasgow Royal Infirmary, Glasgow G31 2ER, UK; Juliet Keidan Institute of Pediatric Gastroenterology, Hepatology and Nutrition, Faculty of Medicine, The Hebrew University of Jerusalem, Jerusalem, Israel; James Watt School of Engineering, University of Glasgow, UK; Department of Gastroenterology, Hospital for Sick Children, University of Toronto, Toronto, Canada; Department of Pediatrics, Gastroenterology and Nutrition, Jagiellonian University Medical College, Cracow, Poland; Division of Clinical and Molecular Medicine, School of Medicine, University of Dundee, Dundee, UK; CHEO Inflammatory Bowel Disease Centre, Division of Gastroenterology, Hepatology and Nutrition, Children’s Hospital of Eastern Ontario, Ottawa, ON K1H 8L1, Canada; Department of Pediatrics, University of Ottawa, Ottawa, ON, Canada; Department of Pediatrics, University of Alberta, Edmonton, AB, Canada; Department of Gastroenterology, Hospital for Sick Children, University of Toronto, Toronto, Canada; Juliet Keidan Institute of Pediatric Gastroenterology, Hepatology and Nutrition, Faculty of Medicine, The Hebrew University of Jerusalem, Jerusalem, Israel; Human Nutrition, School of Medicine, Dentistry & Nursing, University of Glasgow, New Lister Building, Glasgow Royal Infirmary, Glasgow G31 2ER, UK

**Keywords:** microbiome, Crohn’s disease, sequencing, fecal water content

## Abstract

**Introduction:**

We investigated relationships between disease activity measures and the gut microbiome in children with Crohn’s disease (CD) and how these were confounded by gastrointestinal transit time.

**Methods:**

Microbiome was profiled (16S rRNA sequencing) in feces from 196 children with CD. Sixty participants also provided samples after 18 months. Mural inflammation (Pediatric Inflammatory Crohn’s Magnetic Resonance Enterography Index, PICMI), the simple endoscopic score for CD, and the weighted pediatric Crohn’s disease activity index (wPCDAI) were assessed. Fecal calprotectin, plasma C-reactive protein (CRP), and fecal water content (FWC), a proxy of gastrointestinal transit time, were measured too.

**Results:**

Microbiome α diversity, clustering, and differential taxa were related to disease status, but varied remarkably by disease activity measure used. The strongest relationships between microbiome and disease activity status were observed using wPCDAI; fewer or no relationships were seen using more objective measures like PICMI. Taxa predictive of disease activity status were dependent on the disease activity measure used with negligible overlap. Active disease was associated with more pathobionts (eg, Viellonella, Enterobacterales) and fewer fiber-fermenting organisms. The effect FWC had on microbiome superseded the effect of active disease for all disease activity measures, particularly with wPCDAI. Accounting for FWC, the differences in microbial signatures explained by disease activity status were attenuated or lost.

**Conclusions:**

In CD, microbiome signatures fluctuate depending on the measure used to assess disease severity; several of these signals might be secondary disease effects linked with changes in gut motility in active disease. PICMI appears to be less influenced when studying relationships between microbiome and mural inflammation in CD.

**Key Messages**

**- What is already known?**

In Crohn’s disease (CD), gut microbiome is dysbiotic and may be involved in underlying disease pathogenesis.

**- What is new here?**

Relationships between microbiome signatures and disease activity are highly dependent on disease activity measures used and are confounded by gut transit time.

**- How can this study help care?**

This study identified select microbial organisms which may be important for CD pathogenesis and some others whose alterations may be secondary disease effects.

## Introduction

A large body of literature suggests that the gut microbiome plays a crucial role in the pathogenesis of Crohn’s disease (CD), with major compositional shifts observed in patients with CD compared to healthy individuals.^1–3^ Gut microbial composition has been proposed as a potential biomarker for differential diagnosis, prediction of disease outcomes, and response to drug therapy in CD, and as a target for therapeutic intervention.^1,2,4–7^ Previous studies have also explored predictive associations between inflammatory bowel disease (IBD) microbiota signatures and prediction of disease severity.^1^ The selection of measures to assess disease severity has varied among studies, and so it is possible that changes to discrete microbes or sub-communities correlate distinctly with each of these measures. Some of these correlations may be due to confounding and not markers of underlying disease biology. These considerations might be particularly pertinent in CD where illness presents an extensive variation in phenotype, behavior, and alterations in disease biomarkers.^8^

Likewise, it is also possible that these relationships are, at least in part, explained by secondary disease effects and confounded by inflammation itself, changes in gut physiology, luminal environmental changes (for instance, pH and oxygen availability), and motility during active and quiescent disease. Vandeputte et al. have previously shown that stool consistency, indicated by the Bristol Stool Chart, was strongly associated with gut microbiota richness and composition, enterotypes, and bacterial growth rates.^9^ In a population-level analysis of gut microbiome variation in nearly 4000 people, stool consistency showed the largest effect size and superseded the effects diet and certain diseases may have.^10^ It, therefore, becomes imperative to consider the effect of stool consistency and gastrointestinal transit time in microbiome-related research, particularly when the exploration of causal relationships with disease activity markers is the primary study objective of interest.^11^

Thus far, no study has explored how the assessment of disease severity using an array of different disease activity measures (ie, mural, endoscopic, clinical activity, biochemical) relates to the gut microbiome, an environmental factor strongly implicated in underlying disease pathogenesis and progression of CD. This is particularly important now that various disease biomarkers, new tools, and scores have been developed to improve the assessment of disease severity and extent.^12^ Recently, the Pediatric Inflammatory Crohn’s Magnetic Resonance Enterography Index (PICMI) has been developed to assess mural inflammation across the entire gut in children with CD. The PICMI score correlated well with the radiologist’s global assessment and with the simple endoscopic score for CD (SES-CD) in a group of patients with ileocolonic disease.^13^

In this study, we investigated the relationships between various disease activity measures, including the novel PICMI, with the gut microbiome signatures before exploring the extent to which any of these relationships were explained or attenuated by fecal water content (FWC), a proxy measurement of stool consistency and gastrointestinal transit time.

## Materials and Methods

### Recruitment and Sample Collection

This study included samples from pediatric patients with CD who participated in the ImageKids multicentre study.^13^ Eligible participants were children between 5 and 18 years of age with a diagnosis of CD involving any disease phenotype or behavior. Both patients with active disease and those in remission were included. Recruitment was competitive, and there was no limit on the number of participants recruited per site. Participant cumulative enrollment was stratified by disease duration (ie, 20% within 3 months, 20% between 3 months and 2 years, 20% between 2.01 and 3 years, and 40% with disease duration over 3 years). Disease characteristics and medication were collected prospectively from medical notes.

Patients were recruited from Canada, Israel, the United States, Spain, Germany, Poland, the Netherlands, Australia, and Scotland. Data were collected from these participants at study enrollment and for some patients again after 18 months. At baseline, the simple endoscopic score for CD (SES-CD) was calculated during ileocolonoscopy. At both time points, patients provided fecal samples for microbiome analysis and had a magnetic resonance enterography (MRE) assessment and estimation of PICMI score. Overall disease activity was estimated in both visits using the weighted pediatric Crohn’s disease activity index (wPCDAI),^14^ measurements of fecal calprotectin (FCal), and plasma C-reactive protein (CRP).

#### Sample collection

Stool and blood samples were collected from patients at baseline and at 18 months on their follow-up visit. The stool was stored in 200 mg aliquots in sterile tubes and stored at −80 °C. All samples were batch-shipped in dry ice to a central lab for processing and testing. The percentage of FWC was estimated following freeze-drying for 48 h (Edwards Modulyo). Samples with FWC in the first tertile were categorized as low, those in the second tertile were categorized as medium, and those in the third tertile were categorized as high. These categories were used to improve clarity in graphical visualization, with the continuous percentage values used for statistical analyses.

### Disease Severity Classification

#### Mural inflammation and clinical disease activity

Assessment of mural inflammation was performed using MRE at study enrollment and at 18-month follow-up. The validated PICMI score was estimated to evaluate the severity of mural inflammation in children with CD along the entire gastrointestinal tract.^13^ Each time a participant was assigned a PICMI score, they were classified as one of 2 categories: remission (PICMI ≤ 10) or active disease (PICMI > 10). Clinical disease activity was also estimated using the wPCDAI with a score <12.5 classified as remission.^14^

#### Faecal calprotectin and C-reactive protein

CRP was measured at the diagnostic laboratory of each participating site, and FCal centrally (PhiCal Test, Calpro AS, Oslo, Norway). Fecal calprotectin (FCal) and CRP values below 250 mg kg^−1^ and 7 mg L^−1^, respectively, were defined as indicative of biochemical remission. For FCal, cut-off values were set considering the STRIDEII recommendations,^15^ and for CRP we used the upper normal threshold value of the hospital diagnostic laboratories of participating sites.

#### Simple endoscopic score for CD

The SES-CD score was calculated during endoscopy under general anesthesia, and a total score below 3 was considered mucosal healing/remission.^16^

### Microbiome Analysis

Extraction of genomic DNA was performed using the PowerSoil® DNA isolation kit according to the manufacturer’s instructions (Qiagen, Manchester, UK). The V4 region of the 16S rRNA gene was amplified, and sequencing (MiSeq) was carried out on all samples using 2 × 250 bp paired-end reads.

### Bioinformatics

The gut microbiome was represented using an amplicon sequence variant (ASV) table generated using the DADA2^17^ pipeline, version 1.16 (https://benjjneb.github.io/dada2/tutorial.html). Quality filtering was performed with a maximum expected error value for merged sequences of 2, and sequences were truncated at the first instance of a quality score less than 2. The core DADA2 algorithm was applied to remove sequencing noise, forward and reverse sequences were merged, and an ASV table was generated. Chimeras were removed using the DADA2 de novo method, and sequences longer than 256 bp and shorter than 250 bp were filtered out. Amplicon sequence variants were taxonomically classified to genus level against the SILVA 16S reference dataset, release 138,^18^ using the RDP Naive Bayesian Classifier^19^ algorithm (assignTaxonomy in DADA2) with species level added for those with a 100% match to the SILVA dataset using the addSpecies function, as per the DADA2 pipeline.

### Statistics

Statistical analysis was performed using R version 4.1.2^20^ using functions from published R packages in conjunction with scripts coded in-house specifically for use with this dataset. Differences between groups were investigated using Mann–Whitney tests with paired tests used where appropriate, and correlations between variables were assessed using Spearman’s rank correlation test. Associations between the microbiome and potential confounding factors were identified using redundancy analysis following methods outlined by Forrester et al.^21^ Microbiome data were explored, using the vegan version 2.6-4 and phyloseq version 1.44.0 R packages,^22,23^ both in terms of alpha diversity and overall community composition and visualized using non-metric multidimensional scaling (NMDS) of Bray–Curtis distance matrices, evaluated using permutation analysis of variance (ANOVA) implemented in the adonis2 function in vegan with 999 permutations and the identified confounding factors included as covariates (site location, FWC, use of azathioprine and use of biologics). In addition, community variability was assessed using the betadisper function in vegan, which calculates the distance of each sample from its group centroid.

Differences in abundances of individual taxa between groups were identified using the MaAsLin2 version 1.16.0 R package,^24^ with the MaAsLin (Microbiome Multivariable Association with Linear Models) method applied to log-transformed data normalized using total sum scaling, with any confounding factors identified (ie, site location, FWC, use of azathioprine, and use of biologics) included as random effects. We used a more conservative corrected *P* value (*Q* value) of 0.15 than the default 0.25, and corrections for multiple testing were applied using the Benjamini–Hochberg method. Spearman correlation tests were used to find associations among FWC, microbial diversity, and disease severity metrics. Missing data were imputed using the median value of the appropriate variable. The effect of FWC on downstream analysis was investigated by both including and omitting it as a covariate in the previously outlined methods and comparing differences in results.

Due to the absence of a healthy control cohort, microbial dysbiosis (MD) was assessed using results from a previous study by Quince et al. who identified 22 genera that were decreased in CD compared to healthy controls and 14 that were increased.^4^ MD was calculated for each sample separately by taking the natural logarithm of the total sum of reads of increased genera divided by the total sum of reads of decreased genera, as outlined by Gevers et al.^1^

Random forest (RF) analysis was performed using the R package, randomForest,^25^ using ASVs as predictors of disease severity at baseline. The previously defined categories of disease severity for PICMI, FCal, CRP, wPCDAI, and SES-CD were used to generate 5 separate RF models. Variable optimization was applied using the FeatureTerminatoR R package, which minimizes the number of variables in the model without reducing model performance.^26^ For all models, 10,000 decision trees were grown and other input variables were set to default, with repeated 10-fold cross-validation applied to each model. To account for class imbalances, the data were sampled to have the same number of participants for each severity category, and this was refreshed for each individual decision tree in the RF. The importance of each feature in the model was assessed as a mean decrease in the Gini impurity index. A receiver operating characteristic curve (ROC) was plotted, and the area under the curve (AUC) was calculated with the R package ROCR.^27^ Finally, a random subset containing 20% of the samples from the full dataset had been held back and used to test the predictive ability of each model.

## Results

### Patient Population

The gut microbiome from 196 participants was analyzed, of whom 60 participants provided follow-up samples and had assessments after 18 months. Characteristics of both cohorts are presented in Table 1.^13^

**Table 1.** Demographics and clinical data for study participants of both cohorts.

	Baseline (*n* = 196)	Follow-up (*n* = 60)
Country, *n* (%)		
Canada	53 (27)	20 (33)
Israel	53 (27)	16 (27)
USA	32 (16)	6 (10)
Spain	24 (12)	11 (18)
Poland	13 (7)	4 (7)
Germany	10 (5)	1 (2)
Netherlands	4 (2)	1 (2)
Australia	4 (2)	1 (2)
UK (Scotland)	3 (2)	0 (0)
Age at enrollment (years)	14.1 (2.5)	13.9 (2.6)
Male, *n* (%)	111 (57)	39 (60)
Weight (kg)	47.5 (14.8)	48.0 (15.3)
Weight *z*-score	−0.66 (1.4)	−0.59 (1.4)
Height (cm)	157.0 (15.2)	155.9 (15.9)
Height *z*-score	−0.49 (1.2)	−0.57 (1.1)
BMI	18.5 (4.3)	18.8 (4.4)
Disease location, *n* (%)^a^		
L1	44 (23)	11 (18)
L2	32 (16)	8 (13)
L3	114 (58)	40 (67)
L4	4 (2)	1 (2)
Disease behavior, *n* (%)^a^		
B1	138 (71)	44 (73)
B1P	14 (7)	4 (7)
B2	28 (14)	7 (12)
B2P	5 (3)	3 (5)
B2B3	3 (2)	0 (0)
B2B3P	2 (1)	1 (2)
B3	5 (3)	2 (3)
Age at diagnosis, *n* (%)^a^		
A1a: <10 years	54 (28)	19 (32)
A1b: 10-17 years	141 (72)	41 (68)
Medication at enrollment, *n* (%)		
5 Aminosalicylates	26 (13)	9 (15)
Enteral nutrition	25 (13)	7 (12)
Steroids	11 (6)	5 (8)
Methotrexate	42 (22)	12 (20)
Biologics	69 (35)	26 (43)
Azathioprine	39 (20)	12 (20)
Mercaptopurine (6-MP)	10 (5)	6 (10)
Antibiotics	11 (6)	3 (5)
Disease duration, *n* (%)		
0-3 months	48 (24)	13 (22)
3-24 months	39 (20)	13 (22)
24-36 months	29 (15)	7 (12)
>36 months	80 (41)	27 (45)
wPCDAI at enrollment, median (IQR)	25.0 (7.5-42.5, *n* = 175)	22.5 (5.0-45.0, *n* = 57)
FCal at enrollment, median (IQR)	605 (173-1268, *n* = 188)	605 (165-1019, *n* = 59)
Plasma CRP at enrollment, median (IQR)	6.4 (1.9-20.9, *n* = 179)	6.5 (2.6-24.7, *n* = 54)
SES-CD at enrollment, median (IQR)	10 (3.2-16.00, *n* = 190)	6 (3.0-21.5, *n* = 60)

Abbreviations: FCal, fecal calprotectin; SES-CD, simple endoscopic score for Crohn’s disease; wPCDAI, weighted pediatric Crohn’s Disease activity index. Data are presented with a mean (SD) unless it is otherwise stated.

^a^As per Paris classification.

### Baseline Microbiome Diversity Signatures

Using NMDS ordination plots with Bray–Curtis distances, β diversity, a metric of global microbiome composition, was influenced by participants’ country of origin, explaining 3% of its structure variance (*R*^2^ = 0.031, *P* = .001; Supplementary Figure S1).

**Figure 1.**
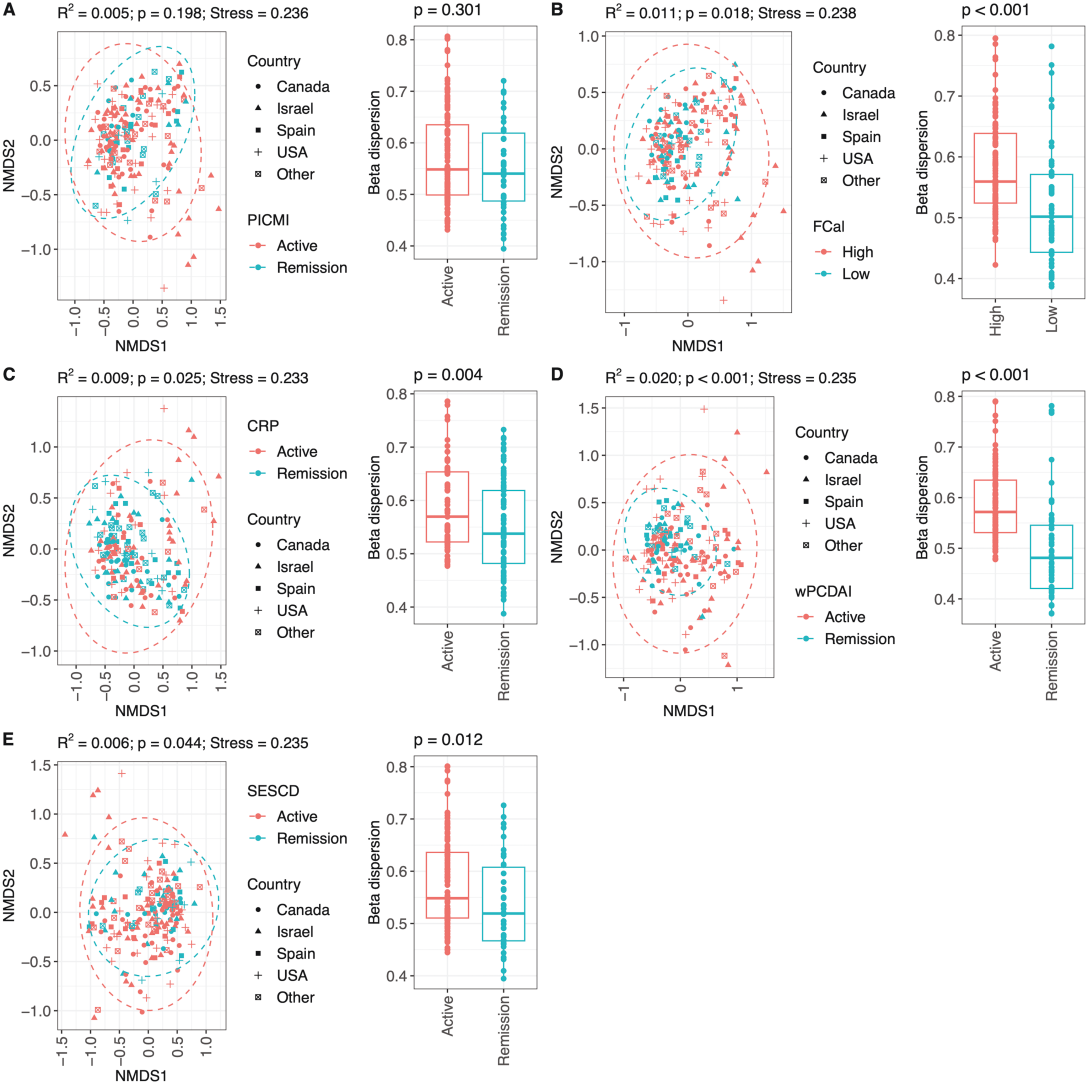
Non-metric multidimensional scaling plots and permutation ANOVA results showing variation in baseline microbiome composition for different disease activity status groups, defined using (A) Pediatric Inflammatory Crohn’s Magnetic Resonance Enterography Index, (B) fecal calprotectin, (C) CRP, (D) weighted pediatric Crohn’s Disease activity index, and (E) simple endoscopic score for Crohn’s disease measurements. Each plot is accompanied by a box plot to show differences in beta dispersion between groups.

Patients were divided into subgroups with active disease or inactive/remission according to each of the 5 disease activity measures (Supplementary Table S1). Disease activity status differed depending on the measure used, but there was significant overlap and correlation between these measures (Supplementary Table S2). Microbiome structure was related to disease activity status but also varied according to the disease activity measure used (Figure 1). While we did not observe any significant differences in β diversity using PICMI (*R*^2^ = 0.005, *P* = .198; Figure 1A) to classify disease activity status, the use of FCal (*R*^2^ = 0.011, *P* = .018; Figure 1B), CRP (*R*^2^ = 0.009, *P* = .025; Figure 1C), wPCDAI (*R*^2^ = 0.020, *P* < .001; Figure 1D), and SES-CD (*R*^2^ = 0.006, *P* = .044; Figure 1E) resulted in significant microbiome differential clustering.

Community variability, measured using beta dispersion, was also not significantly different between PICMI status (*P* = .301, Figure 1A) but was significantly different when FCal (*P* < .001, Figure 1B), CRP (*P* = .004, Figure 1C), wPCDAI (*P* < .001, Figure 1D), and SESCD (*P* = .012, Figure 1E) were used as classifiers of disease status.

Similar patterns were seen when disease activity measures were used as continuous variables. Again, no significant differences were observed using PICMI (*R*^2^ = 0.010, *P* = .098; Supplementary Figure S2A) for classifying disease activity status, but the use of FCal (*R*^2^ = 0.015, *P* < .001; Supplementary Figure S2B), CRP (*R*^2^ = 0.015, *P* < .005; Supplementary Figure S2C), wPCDAI (*R*^2^ = 0.022, *P* < .001; Supplementary Figure S2D), and SES-CD (*R*^2^ = 0.013, *P* = .035; Supplementary Figure S2E) led to significant differential clustering of the microbiome.

**Figure 2.**
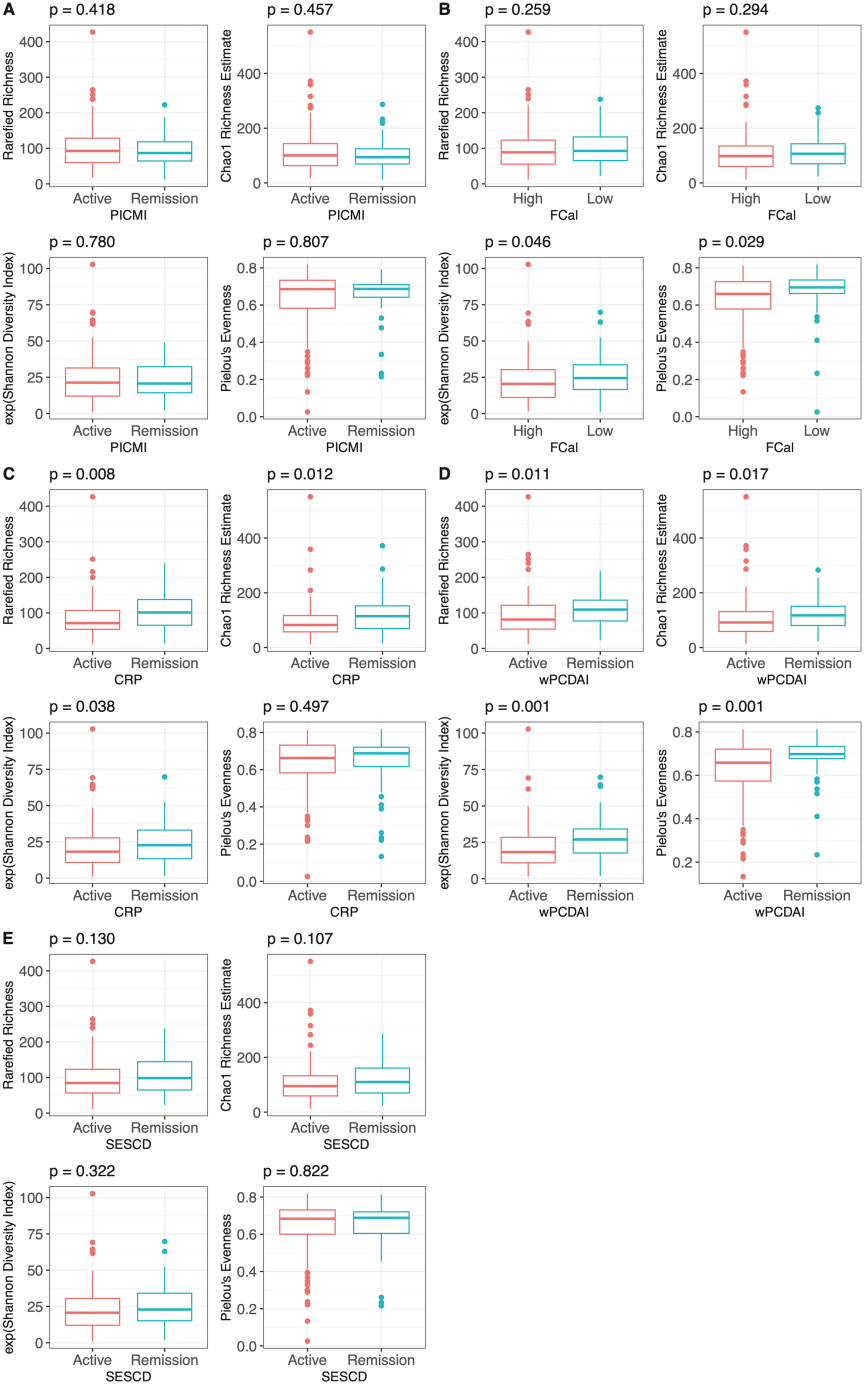
Baseline alpha diversity indices: rarefied richness, Chao1 richness estimate, exp(Shannon diversity), and Pielou’s evenness. Participants were divided into 2 disease activity status groups, defined using (A) Pediatric Inflammatory Crohn’s Magnetic Resonance Enterography Index (B) fecal calprotectin, (C) CRP, (D) weighted pediatric Crohn’s disease activity index, and (E) simple endoscopic score for Crohn’s disease measurements.

Using α diversity estimates, significant differences were identified between disease activity status in rarefied richness (CRP: *P* = .008, wPCDAI: *P* = .011), Chao1 richness estimate (CRP: *P* = .012, wPCDAI: *P* = .017), Shannon diversity index (FCal: *P* = .046, CRP: *P* = .038, wPCDAI: *P* = .001), and Pielou’s evenness (FCal: *P* = .029, wPCDAI: *P* = .001) (Figure 2). In general, active disease was associated with a less diverse microbiome. No differences in α diversity were observed when using PICMI or SES-CD to classify disease activity status. Significant negative correlations between α diversity estimates and disease activity indices used as continuous variables were also detected in rarefied richness (CRP: *r* = −0.23, *P* = .002, wPCDAI: *r* = −0.20, *P* = .008, SESCD: *r* = −0.16, *P* = .032), Chao1 richness estimate (CRP: *r* = −0.21, *P* = .005, wPCDAI: *r* = −0.17, *P* = .022, SES-CD: *r* = −0.16, *P* = .029), Shannon diversity index (CRP: *r* = −0.20, *P* = .006, wPCDAI: *r* = −0.27, *P* = .001, SES-CD: *r* = −0.16, *P* = .028), and Pielou’s evenness (wPCDAI: *r* = −0.26, *P* = .001) (Supplementary Figure S3). None of the α diversity measures correlated significantly with FCal or PICMI.

### Differential Microbiome Signatures Across Disease Activity Measures

**Figure 3.**
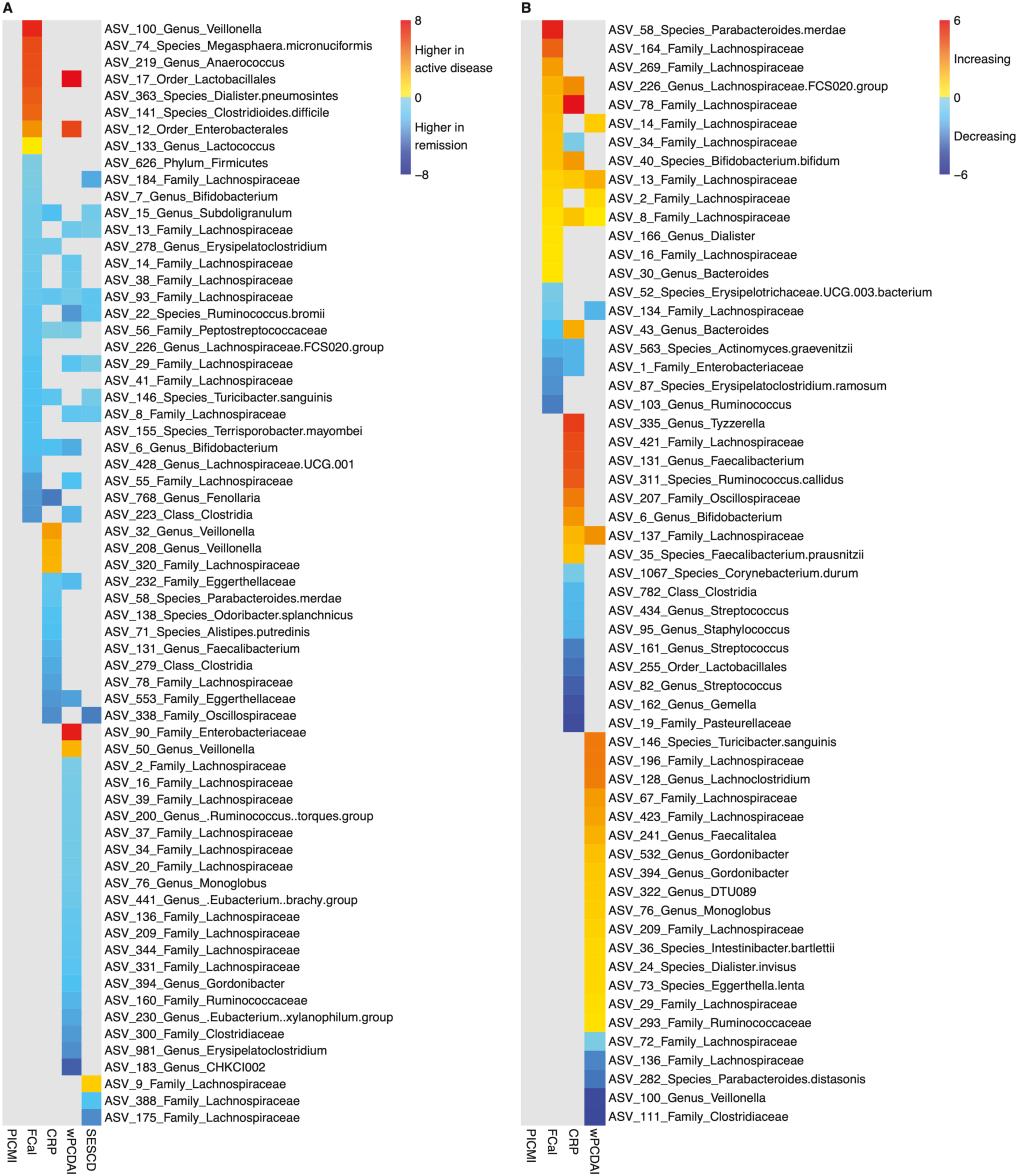
(A) Log2 fold-difference data for significant (*P* < .05) differentially abundant amplicon sequence variants (ASVs) at baseline between participants with active disease and remission, defined using Pediatric Inflammatory Crohn’s Magnetic Resonance Enterography Index (PICMI), fecal calprotectin, CRP, weighted pediatric Crohn’s disease activity index (wPCDAI), and simple endoscopic score for Crohn’s disease measurements. Positive fold differences indicate increased abundance in participants who were in remission. (B) Log2 fold-change data for significant (*P* < .05) differentially abundant amplicon sequence variants for improving patients between baseline (V1) and follow-up (V2). Improving patients were identified as those with improving disease activity status, defined using each of PICMI, fecal calprotectin, CRP, and weighted pediatric Crohn’s disease activity index. Positive fold changes indicate increasing abundance over time.

At study enrollment, no ASVs were significantly different between patients in remission compared with active disease, using the PICMI score (Figure 3A). In contrast, the number of ASVs that were significantly different was 30, 19, 36, and 12 when using FCal, CRP, wPCDAI, and SES-CD, respectively (Figure 3A). When FCal was used to define remission, several ASVs were in high abundance, including ASVs from *Bifidobacterium* and members of *Lachnospiraceae* (Figure 3A). ASVs with higher abundance in remission, such as ASVs 13, 93, 29, 8 (all *Lachnospiraceae*), ASVs 6 and 22 (*Bifidobacterium* and *Ruminococcus bromii*, respectively), along with several other members of Firmicutes, were shared across at least three of the disease activity measures (Figure 3A).

In contrast, ASVs that were in higher abundance in active disease predominantly belonged to recognized pathobionts such as *Enterobacterales* and *Viellonella* as defined by FCal (Figure 3A). *Enterobacterales* was also identified in higher abundance in patients where active disease was determined by wPCDAI (Figure 3A).

Spearman’s rank correlations tests were carried out between individual ASVs and all 5 disease activity measures treated as continuous variables (Supplementary Figure S4). PICMI had the fewest associations with 15 ASVs correlating significantly. In contrast, wPCDAI had the most significant correlations (*n* = 37). These results followed a similar pattern to those from the differential analysis using disease status classification, with recognized pathobionts correlating positively with the severity scores. Specifically, ASVs of the family *Pasteurellaceae* and genus *Veillonella* correlated positively with all disease activity measures, and ASV 12 (*Enterobacterales*) correlated positively with CRP, wPCDAI, and SESCD.

**Figure 4.**
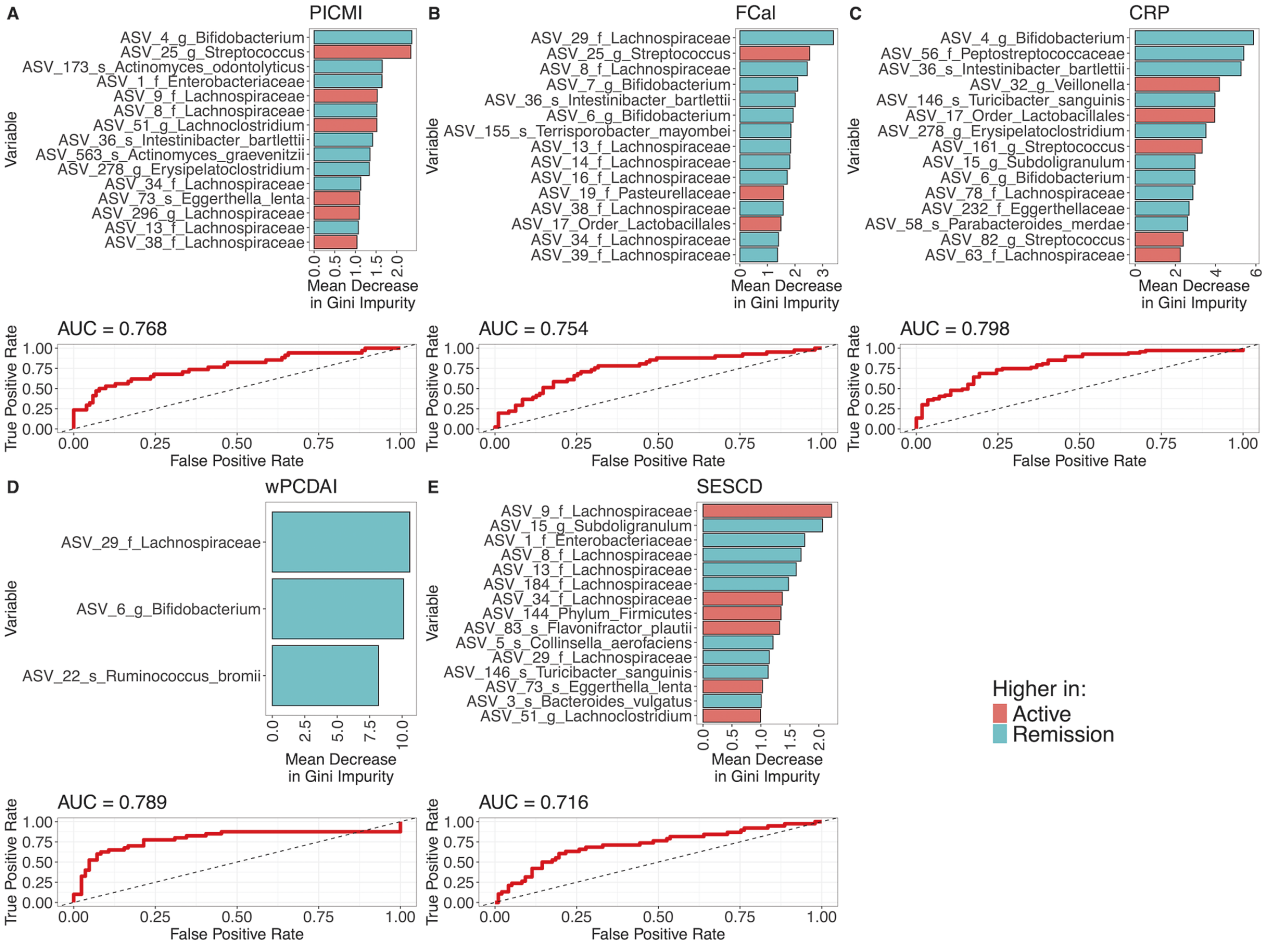
Random forest models using microbiome data to predict disease activity level, defined using (A) Pediatric Inflammatory Crohn’s Magnetic Resonance Enterography Index, (B) fecal calprotectin, (C) CRP, (D) weighted pediatric Crohn’s disease activity index, and (E) simple endoscopic score for Crohn’s disease measurements. The 15 most important amplicon sequence variants, based on a mean decrease in Gini impurity are shown for each model. The receiver operating characteristic curve curve for each model is also shown.

Finally, RF analysis with feature elimination was applied to create models and identify key organisms predictive of disease activity status. Models drawn for all disease activity markers studied were able to differentiate patients with active disease from those in remission statistically better than chance based on out-of-bag (OOB) error rate as well as the error rate when running models on the test subset of the data (Supplementary Table S3, Figure 4). Model performance statistics were in general similar across the various disease activity indices. A microbiome signature was able to classify disease activity status, using PICMI, with the lowest OOB error (21%), the highest specificity (87%), and only modest sensitivity (56%) (Supplementary Table S3). When evaluated using the subset of data held back for testing, the model for prediction of wPCDAI status performed the best with an error rate of 23%. This model also had a low OOB error rate (23%) as well as relatively high specificity (81%) and sensitivity (70%) (Supplementary Table S3). Key ASVs predictive of disease activity status varied significantly across the various disease markers with negligible overlap (Supplementary Figure S5). Of note, 2 ASVs involved in fiber fermentation and short-chain fatty acid production, ASV 22 (*R. bromii*) and ASV 6 (*Bifidobacterium*), along with a third, ASV 29 (*Lachnospiraceae*) were predictive of disease activity status using wPCDAI; all 3 of these were at higher abundance in patients in remission (Figure 4).

### Changes in Microbiome Diversity at Follow-Up

Fecal samples were provided at baseline and follow-up from 60 participants. Patients were divided into 2 groups; patients whose disease activity status improved from active to remission, between baseline and follow-up, and the remainder were classified as non-improved (Supplementary Table S4). As paired endoscopies were not performed, disease improvement using the SES-CD was not computed.

**Figure 5.**
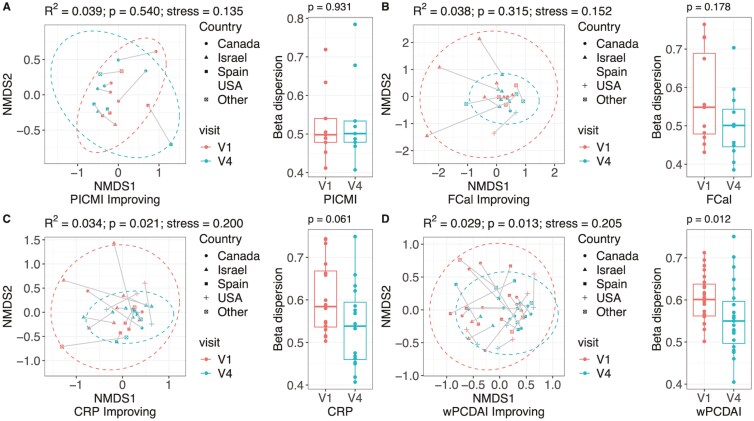
Non-metric multidimensional scaling plots and permutation ANOVA results showing changes in microbiome composition for improving patients between baseline (V1) and follow-up (V2). Improving patients were identified as those with improving disease activity status, defined using (A) Pediatric Inflammatory Crohn’s Magnetic Resonance Enterography Index, (B) fecal calprotectin, (C) CRP, and (D) weighted pediatric Crohn’s disease activity index. Samples from the same participant are linked on each plot. Each plot is also accompanied by a box plot to show differences in beta dispersion between groups.

When viewed using NMDS, no changes in microbiome composition were observed when assessing patient disease status improvement using PICMI or FCal (*R*^2^ = 0.039, *P* = .540, and *R*^2^ = 0.038, *P* = .315; Figure 5A and B). However, microbiome composition changed significantly over time for patients whose disease activity improved based on CRP levels and wPCDAI results (*R*^2^ = 0.034, *P* = .021 and *R*^2^ = 0.029, *P* = .013, respectively; Figure 5C and D). In patients whose disease activity status improved at follow-up, their gut microbiome composition was similar to each other and compared to study enrollment. Beta dispersion was higher at enrollment visit (more severe disease activity) for all disease activity measures except PICMI, but was statistically significant only for wPCDAI (Figure 5D).

Like with β diversity, no significant differences in alpha diversity analysis were observed between the two study timepoints in rarefied richness, Chao1, Shannon’s, or Pielou’s diversity indices when using PICMI (Supplementary Figure S6A) or CRP (Supplementary Figure S6C). When using FCal, the Shannon’s diversity index increased at follow-up (*P* = .045; Supplementary Figure S6B). Shannon’s diversity (*P* = .017) and Pielou’s evenness indices (*P* = .006) also increased in patients whose wPCDAI disease activity classification improved (Supplementary Figure S6D).

### Changes in Taxon Abundance in Patients Whose Disease Activity Status Improved

In total, the abundance of 59 ASVs significantly changed in patients whose disease activity status improved; albeit with little overlap in differentially abundant taxa among the various disease activity measures used. Those that were shared among FCal, CRP, and wPCDAI were increasing in abundance between timepoints and belonged to the Lachnospiraceae family (Figure 3B). Proportionally, the relative abundance of more taxa increased when disease activity improved using wPCDAI and FCal than with CRP classification. Like with study enrollment, the large majority of these ASVs belonged to fiber-fermenting and short-chain fatty acid-producing organisms. In contrast, no taxa changed with disease activity improvement using PICMI scoring (Figure 3B).

Next, this study sought to determine if any organisms increased in improving patients at follow-up were also highly abundant in patients who were in remission at study enrollment by cross-referencing differentially abundant ASVs. Although no ASVs were shared across these datasets and disease activity statuses when using PICMI, we identified a small subset of shared ASVs when using FCal, CRP, and wPCDAI to measure disease activity but not PICMI (Supplementary Table S5). More shared ASVs were identified using wPCDAI measurements. Several of these belonged to the Lachnospiraceae family, and others belonged to genera such as *Gordonibacter, Monoglobus*, and *Turicibacter.*

### The Effect of Gastrointestinal Transit Time on Disease Activity Markers and the Microbiome

**Figure 6.**
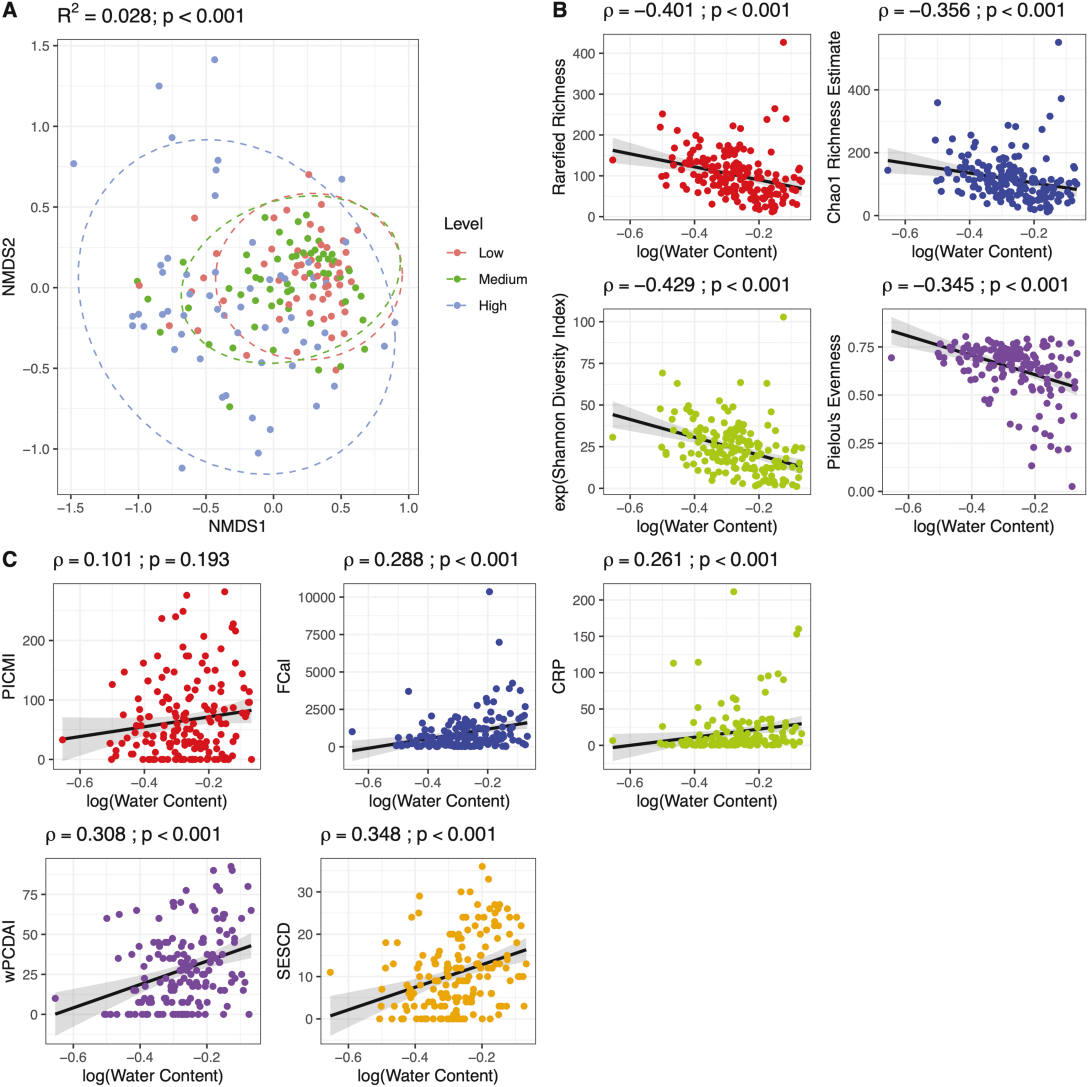
(A) Non-metric multidimensional scaling plot and permutation ANOVA results showing differences in microbiome composition for patients with fecal water content (FWC) levels represented as a categorical variable. (B) Alpha diversity metrics and (C) disease activity scores plotted against FWC. Correlation coefficients (ρ) and *P* values are taken from the results of Spearman correlation tests.

We explored the influence gastrointestinal transit time may have on disease activity markers and the fecal microbiome. The fecal microbiome structure was significantly influenced by FWC content (Figure 6A; *R*^2^ = 0.028 and *P* = .001). The degree of variance in microbiome structure explained by FWC superseded the disease activity status effects reported for all disease activity markers. FWC correlated negatively with microbiome rarefied richness (*P* < .001), Chao1 richness estimate (*P* < .001), Shannon diversity (*P* < .001), and Pielou’s evenness (*P* < .001) (Figure 6B). Among the various disease activity markers, FCal (*P* < .001), CRP (*P* = .001), wPCDAI (*P* < .001), and SES-CD (*P* < .001) were also all positively correlated with FWC, whereas PICMI was not (Figure 6C).

**Figure 7.**
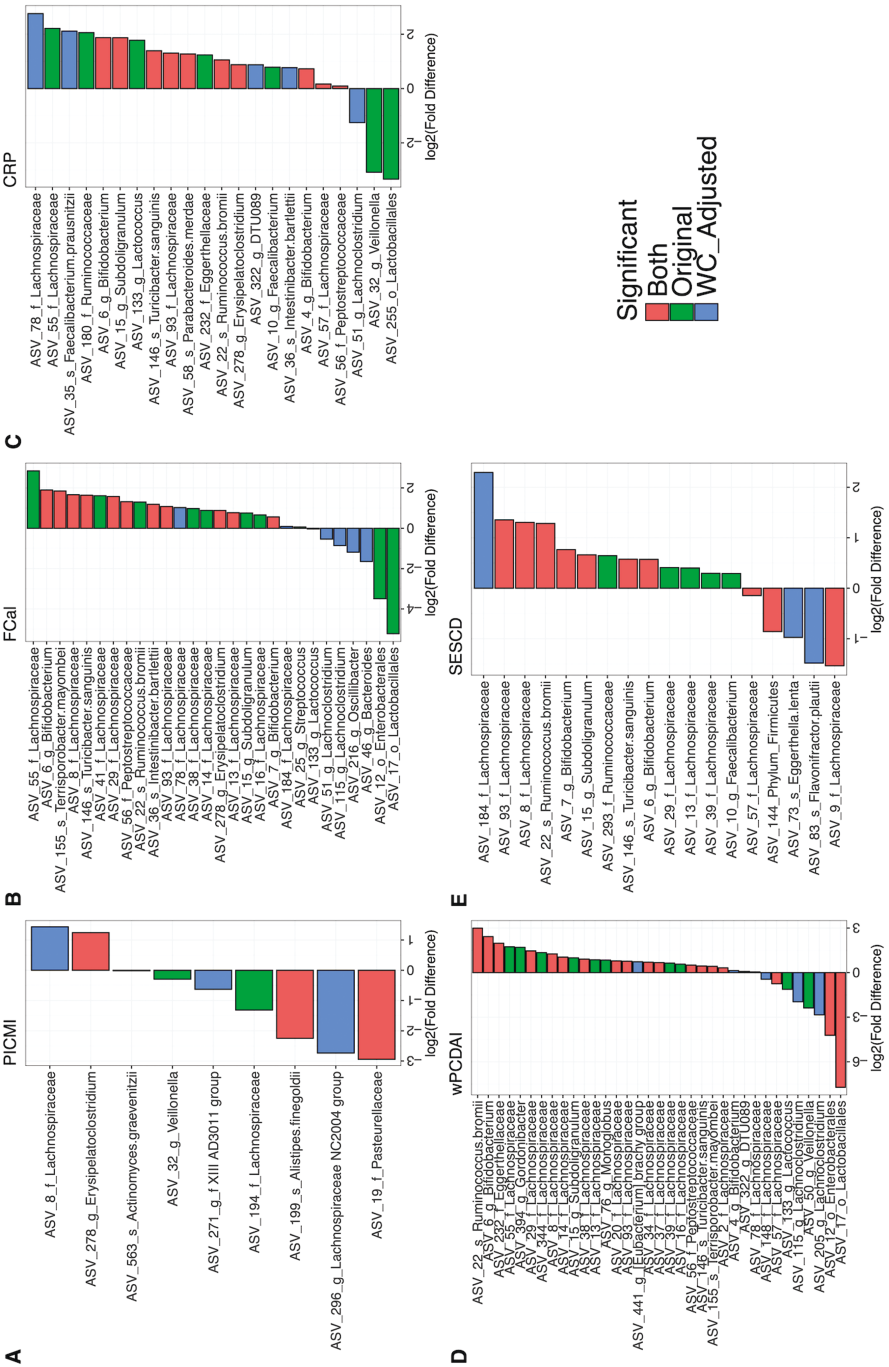
Significant (*P* < .05) differentially abundant amplicon sequence variants (ASVs) at baseline between participants with active disease and remission, defined using (A) Pediatric Inflammatory Crohn’s Magnetic Resonance Enterography Index, (B) Fecal calprotectin, (C) CRP, (D) weighted pediatric Crohn’s disease activity index, and (E) simple endoscopic score for Crohn’s disease measurements. Positive fold differences indicate increased abundance in participants who were in remission. Bars are colored to show if ASVs were significant when corrected or not for fecal water content (FWC) levels. “Original” denotes significance only before accounting for FWC; “WC_adjusted” denotes significance only after accounting for FWC; “Both” denotes significance both before and after accounting for FWC.

Upon observing the impact that FWC has on the microbiome signatures associated with each disease activity measure, microbiome analyses were repeated to investigate if correcting for FWC would modify the microbiome signals observed at all participant samples collected at study enrollment. Accounting for FWC in analysis, the proportion of microbiome structure variance and alpha diversity metrics explained by patients’ disease activity status was attenuated or lost (Supplementary Table S6). Except for PICMI, for all other disease activity measures, the total number of differentially abundant ASVs decreased, following correction of FCW, including ASVs with a large mean size effect prior to FWC adjustment (Figure 7A–D). Several differential ASVs were lost, but few others became statistically significant following FWC adjustment.

### Association Between MD And Disease Severity

All disease activity metrics, PICMI (*P* < .001), FCal (*P* = .001), CRP (*P* < .001), wPCDAI (*P* < .001), and SES-CD (*P* = .010) were positively correlated with MD (Supplementary Figure S7A–E). Non-metric multidimensional scaling ordination using Bray–Curtis distances depicted outliers in terms of microbial community composition as having higher MD, and permutation ANOVA results showed that this was significant (*R*^2^ = 0.023, *P* < .001, Supplementary Figure S7F).

## Discussion

This study explored relationships between fecal microbiome signatures and various disease activity measures in pediatric CD. We hypothesized that key groups of organisms likely to be associated causally with CD development would be independent of the marker used to assess disease status and that different organisms would relate with the various phenotypic presentations of CD. We also assumed that several of the microbial signals observed would be secondary to disease effects, in particular, confounded by changes in gastrointestinal physiology and in FWC a proxy of gastrointestinal transit time.

Differential analysis identified several ASVs that differed between patients with active disease and those in remission. In accordance with previous research, we found higher levels of Lachnospiraceae and beneficial *Bifidobacterium* in patients in remission and observed that MD correlated positively with all disease activity measures.^1,3^ Members of Lachnospireceae include key producers of short-chain fatty acids from fiber fermentation, like butyrate, which is an important energy substrate for colonocytes implicated in the maintenance of intestinal health. In contrast, putative pathogenic and pathobiont organisms from *Pasteurella*, *Veillonella*, *Actinomyces*, and Enterobacteriales were associated with active disease; findings supported by previous research.^1,3^ In longitudinal analysis, the microbial signatures of patients in remission at study enrollment resembled those of patients whose disease status improved at follow-up, hence offering additional confidence that such organisms are potentially important in underlying disease pathogenesis. Collectively, the microbiome features observed here represent the typical characteristics of MD seen in patients with active CD and in remission; typically reduced α diversity, depletion of beneficial organisms, and a parallel increase in the abundance of pathogenic organisms and pathobionts.^1,3^

Against our expectations, far more ASVs associated with disease activity status assessed by wPCDAI than any other disease activity measure. Mural inflammation, assessed by the validated PICMI, produced the fewest relationships, both in the cross-sectional as well as in the prospective longitudinal study analysis. Few organisms associated with disease activity were shared between the various disease activity measures too. This was predominantly observed when stratifying disease activity by FCal, CRP, and wPCDAI, but hardly any shared ASVs were identified when using PICMI. These variable relationships between disease activity measures and microbiome signatures could, in principle, represent causal relationships between specific organisms with the variable phenotypic characteristics of active CD. Thus, different organisms may be implicated in mural inflammation or with gut inflammation limited to the mucosa, as indicated by SES-CD, or with neutrophil infiltration at the gut, as indicated by FCal levels. Alternatively, they may be the result of reverse causation. Trying to unravel such complex relationships is not a trivial effort and one we are currently unable to do within the boundaries of observational data.

Interestingly, the PICMI, in contrast to all other disease activity measures, did not correlate with FCW, indicating that PICMI, a marker of mural inflammation, is potentially less influenced by gastrointestinal transit time, a factor that should be, but rarely is, accounted for in gut microbiome studies.^11^ By extension, this means that relationships established between microbial signatures and disease activity measures of mural inflammation, such as the PICMI, might be less confounded by the profound effect gastrointestinal transit time has on the microbiome; a finding observed here as well as in previous research.^11^ In support of this suggestion is our finding that when correcting for FWC, the differences in α diversity indices or the proportion of variance in microbiome composition explained by disease activity status was attenuated significantly, or, in some cases, statistical significance disappeared. This was seen with regards to FCal, CRP, wPCDAI, and SES-CD but not with PICMI. These effects were carried over to differentially abundant taxon analysis where the total number of ASVs in PICMI-determined remission and active disease remained unchanged, whereas the number of differentially abundant taxa decreased between disease activity status determined by FCal, CRP, and wPCDAI. Interestingly, this effect was more profound for disease activity status assessed using wPCDAI, a modest proxy of gut inflammation, which nonetheless presented the strongest relationships with microbial signatures. Since wPCDAI is highly dependent on gastrointestinal symptoms it might not be surprising we identify more significant relationships with all microbiome parameters studied in the current study.

With the exception of PICMI, the relative abundance of aerotolerant members of *Lactobacillus, Enterobacteriales*, and *Lactococcus* was higher in patients with active disease, whereas anaerobes, like *Bifidobacterium* and members of Lachnospiraceae, a major group of fiber fermenters, were observed at much lower abundance. Gut inflammation is a major modifier of microbiome composition and function, and it is possible that the microbial signals with disease activity status we observed here reflect the relative high oxygen tension in inflamed tissue. In previous analysis of intestinal microbiome in animals and humans, luminal oxygen levels and nutrients provided by host tissue had major effects on gut microbiome composition.^28^ Further evidence to corroborate our speculation that several of the features of MD seen in the CD are secondary disease effects, comes from clinical interventions where attempts to increase the abundance of fiber-fermenting species with prebiotic/fiber supplementation to induce normobiosis using fecal material transfer and beneficial probiotics, failed to show a positive signal.^3^

Taken together, these findings strengthen our conclusions that PICMI is perhaps better suited to exploring causal interactions between the gut microbiome and mural-specific inflammation severity, given the associations found seem to be more independent of the influence of FWC in contrast to all other disease activity measures analyzed. Although fewer organisms were associated with PICMI, it is possible that these organisms (the majority of which were known pathobionts previously linked to active disease) are important candidates to consider in the initiation and propagation of mural inflammation within mechanistic investigations. We also believe that in future fecal microbiome research, it is imperative that researchers account for the effect gastrointestinal transit time may have on microbiome signals, simply using measurements of FWC or the Bristol Stool Chart score, analogous to accounting for disease parameters and concomitant drug therapies.

The current study has several strengths, including the recruitment of a large international cohort of children with CD and the use of a wide range of disease activity indices, including the novel validated PICMI, which requires further independent validation. An important limitation is incomplete sampling, limiting the number of paired samples available for analysis from the longitudinal cohort, and the small number of patients whose disease deteriorated at follow-up to allow us to study better whether microbial signals related when disease improved reverted in patients whose disease deteriorated. The conclusions of this study are also only applicable to the fecal microbiome, and it is possible that different effects are observed in the mucosal adherent microbiome that may have a more protagonist role in the pathogenesis of CD.

In conclusion, this study demonstrated that microbiome signatures in CD fluctuate depending on the measure used to assess disease activity. Future studies should consider both the variability inherent to the various disease activity measures and the influence variables like FWC and gastrointestinal transit time have on microbiome signatures. PICMI appeared to be less influenced, therefore, may make it more suitable to explore the relationship between microbiome and mural-specific inflammation in CD.

## Supplementary Data

Supplementary data is available at *Inflammatory Bowel Diseases* online.

izae199_suppl_Supplementary_Material

## Data Availability

Sequencing data will become available on European Nucleotide Archive, accession number PRJEB76405.

## Study Registration

https://clinicaltrials.gov/study/NCT01881490.
