# Supplementary material for: Gut Microbial Signatures in Pediatric Crohn’s Disease Vary According to Disease Activity Measures and Are Influenced by Proxies of Gastrointestinal Transit Time: An ImageKids Study
Source: Inflamm Bowel Dis. 2024 Oct 17;31(6):1616–29. doi: 10.1093/ibd/izae199 (PMC12166297; doi:10.1093/ibd/izae199)
Supplement: izae199_suppl_Supplementary_Material [file izae199_suppl_supplementary_material.zip › izae199_suppl_Supplementary_Tables_1-6.docx]

**Supplementary Figure Legends
SFigure 1:** NMDS plot and permutation ANOVA results showing baseline differences in microbiome composition for participants whose baseline samples were collected in different countries**.**

**SFigure 2:** NMDS plots and permutation ANOVA results showing variation in baseline microbiome composition for continuous (A) PICMI, (B) Faecal Calprotectin, (C) CRP, (D) wPCDAI, and (E) SES-CD disease activity measurements.

**SFigure 3:** Spearman correlation results between each disease activity measurement: (A) PICMI, (B) Faecal Calprotectin, (C) CRP, (D) wPCDAI, and (E) SES-CD and alpha diversity indices: rarefied richness, Chao1 richness estimate, exp(Shannon diversity), and Pielou’s evenness.

**SFigure 4:** Spearman correlation results between each disease activity measurement: (A) PICMI, (B) Faecal Calprotectin, (C) CRP, (D) wPCDAI, and (E) SES-CD and individual ASVs.

**SFigure 5:** Venn diagram displaying overlap of influential ASVs by disease activity marker and status following Random Forest analysis.

**SFigure 6:** Alpha diversity indices: rarefied richness, Chao1 richness estimate, exp(Shannon diversity), and Pielou’s evenness for improving patients between baseline (V1) and follow-up (V2). Improving patients were identified as those with improving disease activity status, defined using (A) PICMI, (B) Faecal Calprotectin, (C) CRP, and (D) wPCDAI. Samples from the same participant are linked on each plot.

**SFigure 7:** Spearman correlation results between each disease activity measurement: (A) PICMI, (B) Faecal Calprotectin, (C) CRP, (D) wPCDAI, and (E) SES-CD and microbial dysbiosis index. (F) NMDS plot and permutation ANOVA results showing variation in baseline microbiome composition with microbial dysbiosis index.

**Supplementary Tables**

**STable 1:** Number of patients with disease activity status at baseline, defined using PICMI, faecal calprotectin, CRP, wPCDAI, and SES-CD.

|  | **Remission/Low** | **Active/High** |
| --- | --- | --- |
| PICMI, n | 46 | 150 |
| Faecal calprotectin, n | 60 | 129 |
| CRP, n | 94 | 86 |
| wPCDAI, n | 56 | 120 |
| SESCD, n | 47 | 121 |

**STable 2:** Correspondence between activity measures at baseline. Categorical variables are described in columns 1 to 3. Spearman’s rank correlation coefficient is calculated using the continuous versions of the variables.

|  | **Shared (Remission/Low)** | **Shared (Active/High)** | **Different** | **Correlation Coefficient** |
| --- | --- | --- | --- | --- |
| PICMI vs. FCal | 25 | 111 | 53 | 0.299 (p < 0.001) |
| PICMI vs. CRP | 33 | 77 | 70 | 0.282 (p < 0.001) |
| PICMI vs. wPCDAI | 21 | 100 | 75 | 0.421 (p < 0.001) |
| PICMI vs. SESCD | 20 | 102 | 41 | 0.378 (p < 0.001) |
| FCal vs. CRP | 45 | 76 | 52 | 0.329 (p < 0.001) |
| FCal vs. wPCDAI | 37 | 103 | 29 | 0.304 (p < 0.001) |
| FCal vs. SESCD | 30 | 100 | 29 | 0.408 (p < 0.001) |
| CRP vs. wPCDAI | 41 | 66 | 57 | 0.483 (p < 0.001) |
| CRP vs. SESCD | 29 | 62 | 58 | 0.204 (p = 0.013) |
| wPCDAI vs. SESCD | 23 | 89 | 38 | 0.416 (p < 0.001) |

**STable 3:** Performance statistics for random forest models predicting disease activity level at baseline, defined using PICMI, faecal calprotectin, CRP, wPCDAI, and SESCD. ‘Positive’ relates to remission/low and ‘negative’ relates to active/high.

|  | **PICMI** | **Faecal Calprotectin** | **CRP** | **wPCDAI** | **SESCD** |
| --- | --- | --- | --- | --- | --- |
| Specificity (%) | 87 | 78 | 70 | 81 | 80 |
| Sensitivity (%) | 56 | 59 | 75 | 70 | 58 |
| Positive Predictive Value (%) | 59 | 53 | 75 | 64 | 54 |
| Negative Predictive Value (%) | 86 | 81 | 70 | 85 | 83 |
| Out of Bag Error Rate (%) | 21 | 28 | 27 | 23 | 26 |
| Area Under ROC Curve | 0.768 | 0.754 | 0.798 | 0.789 | 0.716 |
| Test Dataset Error Rate (%) | 30 | 27 | 43 | 23 | 40 |

**STable 4:** Number of patients with change in disease activity status between baseline and follow-up, defined using PICMI, faecal calprotectin, CRP, and wPCDAI.

|  | **Improving** | **Not Improving** |
| --- | --- | --- |
| PICMI, n | 9 | 51 |
| Faecal calprotectin, n | 12 | 35 |
| CRP, n | 16 | 37 |
| wPCDAI, n | 23 | 28 |

**STable 5:** For each method of categorising disease activity, ASVs which were significantly differentially abundant between disease activity status groups at baseline and also significantly changed in improving participants between baseline and follow-up.

| PICMI | FCal | CRP | wPCDAI |
| --- | --- | --- | --- |
|  | ASV_8 Lachnospiraceae | ASV_6 Bifidobacterium | ASV_146 Turicibacter sanguinis |
|  | ASV_14 Lachnospiraceae |  | ASV_13 Lachnospiraceae |
|  |  |  | ASV_394 Gordonibacter |
|  |  |  | ASV_322 DTU089 Lachnospiraceae |
|  |  |  | ASV_76 Monoglobus |
|  |  |  | ASV_29 Lachnospiraceae |

**STable 6:** P-values for α diversity metrics and permutation ANOVA analysis to show differences between disease activity groups for each method of classification and before/after correction for faecal water content level. R^2^ values are also shown for permutation ANOVA results.

| **Method** | **%FWC Correction (Before/After)** | **Alpha diversity p-value** | | | | **Permutation ANOVA** | |
| --- | --- | --- | --- | --- | --- | --- | --- |
|  |  | Rarefied Richness | Chao1 | exp(Shannon Diversity) | Pielou's Evenness | R^2^ | P-value |
| PICMI | *Before* | 0.418 | 0.457 | 0.781 | 0.807 | 0.60% | 0.226 |
|  | *After* | 0.501 | 0.476 | 0.815 | 0.763 | 0.70% | 0.219 |
|  |  |  |  |  |  |  |  |
| Fcal | *Before* | 0.259 | 0.294 | 0.046 | 0.029 | 1.30% | 0.003 |
|  | *After* | 0.936 | 0.987 | 0.423 | 0.338 | 1.10% | 0.013 |
|  |  |  |  |  |  |  |  |
| CRP | *Before* | 0.008 | 0.012 | 0.038 | 0.497 | 1.00% | 0.024 |
|  | *After* | 0.029 | 0.043 | 0.155 | 0.858 | 0.90% | 0.108 |
|  |  |  |  |  |  |  |  |
| wPCDAI | *Before* | 0.011 | 0.017 | 0.001 | 0.001 | 2.40% | 0.001 |
|  | *After* | 0.451 | 0.496 | 0.153 | 0.123 | 1.70% | 0.002 |
|  |  |  |  |  |  |  |  |
| SES-CD | *Before* | 0.113 | 0.095 | 0.261 | 0.808 | 0.80% | 0.027 |
|  | *After* | 0.947 | 0.860 | 0.948 | 0.507 | 0.76% | 0.145 |
